# Heart rate as a proxy for estimating oxygen consumption rates in loggerhead turtles (*Caretta caretta*)

**DOI:** 10.1242/bio.058952

**Published:** 2022-03-31

**Authors:** Chihiro Kinoshita, Ayaka Saito, Kentaro Q. Sakamoto, Yasuaki Niizuma, Katsufumi Sato

**Affiliations:** 1 International Coastal Research Center, The Atmosphere and Ocean Research Institute, The University of Tokyo, 1-19-8 Akahama, Otsuch, Iwate 028-1102, Japan; 2 Department of Marine Bioscience, Atmosphere and Ocean Research Institute, The University of Tokyo, 5-1-5 Kashiwanoha, Kashiwa, Chiba 277-8564, Japan; 3 Faculty of Agriculture, Meijo University, 1-501 Shiogamaguchi, Tenpaku-ku, Nagoya, Aichi 468-8502, Japan

**Keywords:** Heart rate, Oxygen consumption rate, Metabolic rate, Sea turtle, Diving response, Marine reptile

## Abstract

Heart rates of air-breathing diving animals can change on a short time scale due to the diving response during submergence. Heart rate is used frequently as a proxy for indirectly estimating metabolic rates on a fine time scale. However, most studies to date have been conducted on endothermic diving animals, and the relationships between metabolic rates and heart rates in ectothermic diving animals have not been well studied. Sea turtles are unique model organisms of diving ectotherms because they spend most of their life in the ocean and perform deep and/or long dives. In this study, we examined the relationship between heart rates and metabolic rates in captive loggerhead turtles, *Caretta caretta*, to estimate oxygen consumption rates during each dive based on heart rates. The oxygen consumption rates (*V̇*_O2_: mlO_2_ min^−1^ kg^−1^) and average heart rates (*f*_H_: beats min^−1^) were measured simultaneously in indoor tanks at water temperatures of 15–25°C. Our results showed that oxygen consumption rate was affected by heart rate and water temperature in loggerhead turtles. Based on the collected data, we formulated the model equation as *V̇*_O2_=0.0124*f*_H_+0.0047*T*_w_ - 0.0791. The equation can be used for estimating fine-scaled field metabolic rates in free-ranging loggerhead turtles. The results of this study will contribute to future comparative studies of the physiological states of ectothermic diving animals.

## Abstract (precis)

**Summary:** The relationship between oxygen consumption rate and heart rate in the loggerhead turtle.

## INTRODUCTION

Energy is an important ecological currency which influences growth rate, reproduction, and survival of animals through behavioural decisions, and it is essential to maximise fitness (Brown et al., 2004; Butler et al., 2004; Tomlinson et al., 2014; Wilson et al., 2020). Wild animals alternate between behaviours (e.g. resting, foraging, and travelling) on short time scales, thus these behavioural traits must be explained by quantitative energy budgets (Speakman, 1997). However, only few methods have been developed for measuring field energy expenditure on a fine-scale in marine vertebrates such as air-breathing diving animals (e.g. Butler et al., 2004; Green, 2011; Williams and Maresh, 2015).

Field metabolic rates of large marine vertebrates have been measured using direct and indirect methods. The doubly labelled water technique is a direct method to assess time-averaged metabolic rates (Hicks et al., 2020; Shirai et al., 2015; Speakman, 1997). Other methods based on dynamic accelerations can indirectly estimate metabolic rates for marine vertebrates (Gleiss et al., 2010; Halsey et al., 2011; Jeanniard-du-Dot et al., 2017). As electrocardiogram (ECG) loggers allow us to obtain fine-scale (from minutes to days) heart rate profiles over the long term (days in Southwood et al., 1999; weeks in Boyd et al., 1999; months in Halsey et al., 2010; more than 1 year in Green et al., 2009), this method is suitable for estimating energy expenditure in wild aquatic vertebrates. However, the relationship between heart rate and metabolic rate varies in some aquatic vertebrates depending on behaviours, ambient temperature, and physiological state (Butler et al., 2002; Froget et al., 2001; Ward et al., 2002). For example, in king penguins (*Aptenodytes patagonicus*), the oxygen consumption rate increases more with an elevated heart rate at rest in low temperatures than during exercise (Froget et al., 2001). By contrast, there was no significant difference in the relationship between running on land and swimming underwater in gentoo penguins (*Pygoscelis papua*) (Bevan et al., 1995). Air-breathing animals show bradycardia and tachycardia during dive, and blood flow to organs and tissues changes during submergence (Zapol et al., 1979). It is unclear how oxygen consumption rates correspond immediately with heart rates. Thus, calibration of the relationship between heart rate and metabolic rate in advance is essential when using heart rate as a proxy of energy expenditure for diving animals (Butler et al., 2004; Green, 2011).

The loggerhead turtle (*Caretta caretta*, Linnaeus, 1758), a species in the family Cheloniidae, is a marine reptile that inhabits offshore and neritic habitats and spends most of its time diving (Lutz and Bentley, 1985; Hatase et al., 2007; Hawkes et al., 2011; Hochscheid et al., 2005; Narazaki et al., 2015). According to tracking studies using satellite relay data loggers, loggerhead turtles perform deep (>200 m) and long (>5 h) dives (Broderick et al., 2007; Hatase et al., 2007; Hawkes et al., 2007; Hochscheid et al., 2005, 2007; Narazaki et al., 2015). They forage, rest, and travel during dives (Houghton et al., 2002; Kinoshita et al., 2021; Minamikawa et al., 2000; Narazaki et al., 2013), and their heart rate decreases during dives or inactive phases (Hochscheid et al., 2002; Sakamoto et al., 2021; Williams et al., 2019). Marine iguanas, sea snakes, and saltwater crocodiles, which mainly inhabit shallow water areas, are also diving reptiles; however, they rarely dive as deep and long as sea turtles (Cook et al., 2016; Heatwole and Seymour, 1975; Rubinoff et al., 1986; Seebacher et al., 2005; Wikelski and Trillmich, 1994). Therefore, sea turtles are physiologically unique reptiles that are particularly adapted to deep and long diving. Measurements of fine-scale field energy expenditure are physiologically and ecologically important for interpreting behaviour in sea turtles, however, no studies on quantitative measurement of field energy expenditures have been conducted in loggerhead turtles so far. According to previous studies on air-breathing diving animals, including marine reptiles (Goldbogen et al., 2019; Southwood et al., 1999; Williams et al., 2017), heart rate is a robust indicator for understanding physiological states, such as the diving response and energy expenditure, on a fine-scale.

Regarding metabolic rates, some previous studies have measured oxygen consumption rates in captive loggerhead turtles (Hochscheid et al., 2004; Kinoshita et al., 2018; Lutcavage et al., 1987; Lutz and Bentley, 1985; Lutz et al., 1989), and studies on sea turtles heart rates have also been conducted using captive animals (Hochscheid et al., 2002; Okuyama et al., 2020; Sakamoto et al., 2021; Southwood et al., 2003; Williams et al., 2019). Field measurements of sea turtle heart rates have only been conducted in the breeding areas of leatherback turtles (*Dermochelys coriacea*) (Southwood et al., 1999). These studies revealed that diving heart rates show a 30% reduction from the surface. Most methods for measuring heart rates require an invasive approach such as electrode insertion, which induces lengthy handling stress (Okuyama et al., 2020). Recently, a non-invasive method based on attaching an ECG electrode patch on the carapace was developed for hard-shelled sea turtles (Kinoshita et al., 2022; Sakamoto et al., 2021). This method facilitates measuring heart rates under natural conditions without an invasive approach. Thus, by combining the methods for measuring oxygen consumption rates (Kinoshita et al., 2018) with non-invasive methods for measuring heart rates, it is possible to examine the relationship between oxygen consumption rates and heart rates in loggerhead turtles with less handling stress.

In this study, we simultaneously measured the oxygen consumption rates and heart rates of loggerhead turtles at various water temperatures and assessed the relationships of these parameters. In addition, we assessed whether heart rates could be used as a proxy for oxygen consumption rates.

## RESULTS

**Fig. 1.**
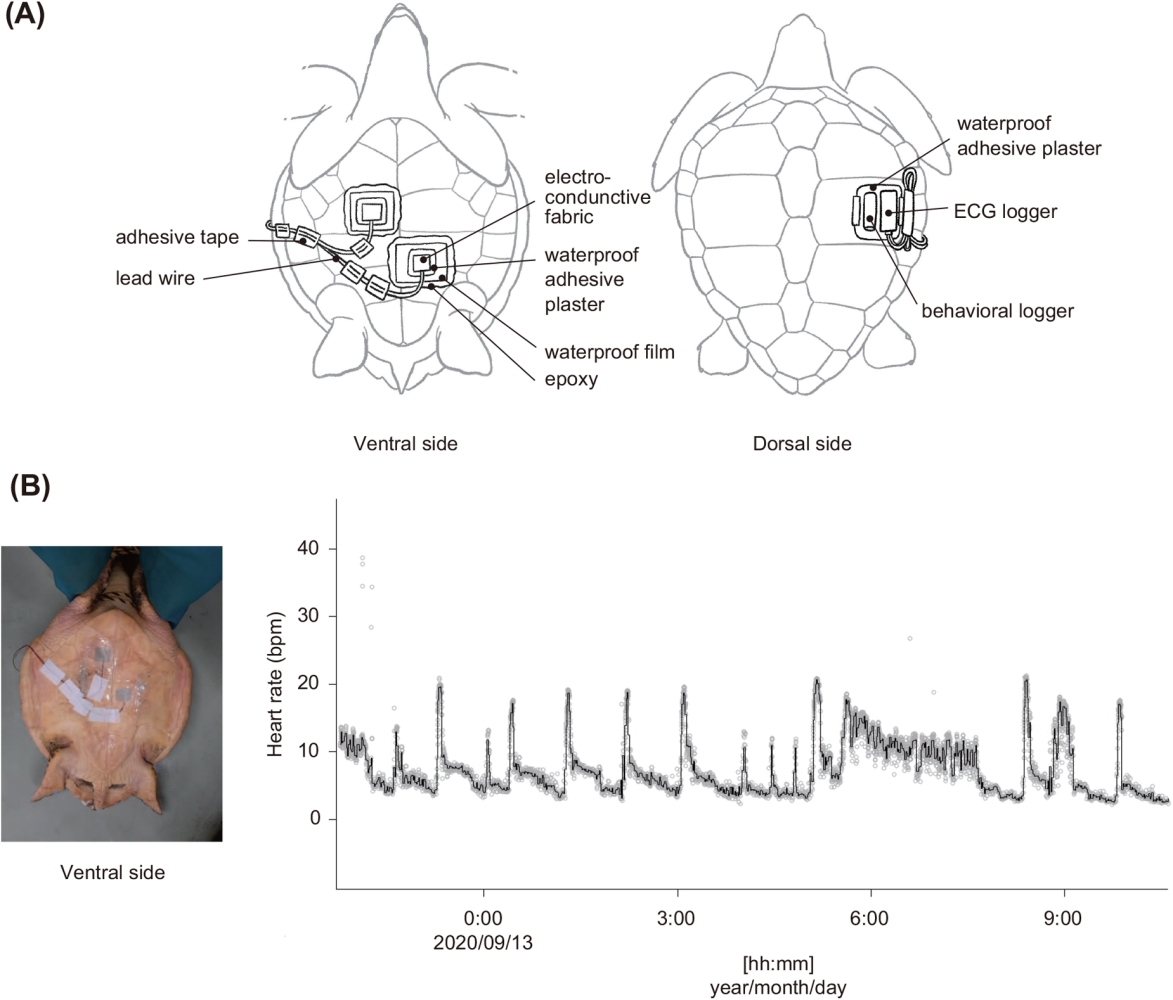
**(A) A loggerhead turtle deployed with an ECG recorder and accelerometer and (B) the example of time-series data of heart rate.** The time-series graph indicates the heart rate when the ECG patches were attached on the ventral side. The black line and grey dots indicate heart rate per minute and instantaneous heart rate, respectively.

**Fig. 2.**
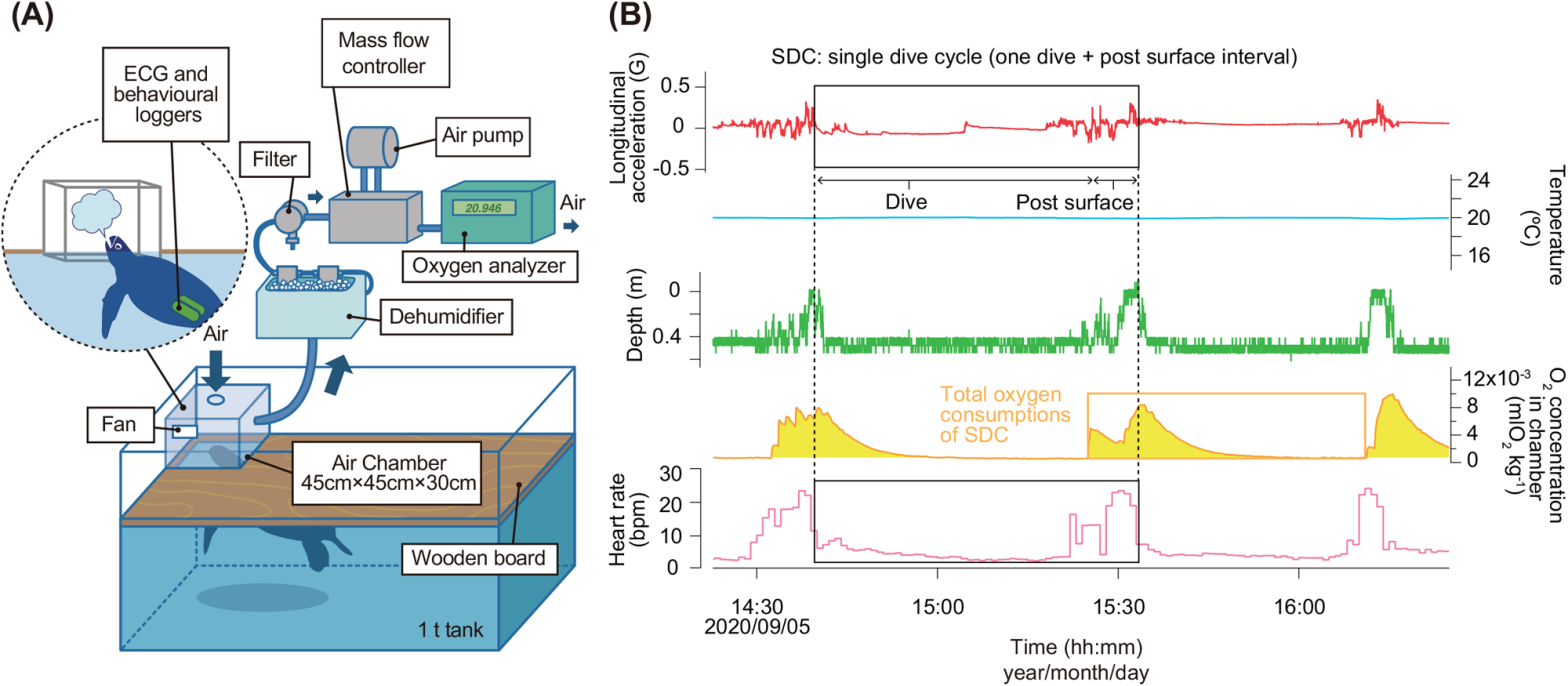
**(A) Experimental design for the respirometry system (see** **Kinoshita et al., 2018****).** (B) Example of time-series data during respirometric measurement from a loggerhead turtle (L2016). The red, blue, green, orange, and pink lines indicate longitudinal dynamic acceleration, water temperature, depth, oxygen consumption and heart rate, respectively. The black squares and dotted lines indicate an SDC, including one dive and post surface interval. The orange area represents total oxygen consumption of SDC.

**Fig. 3.**
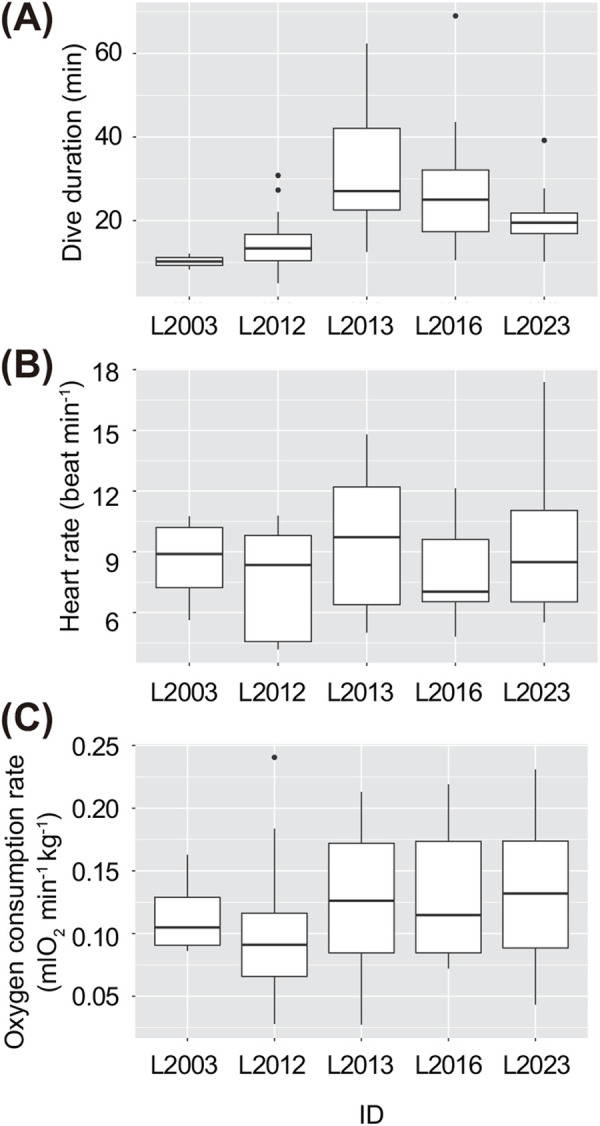
Box plots showing (A) dive durations, (B) heart rates, and (C) oxygen consumption rates of five loggerhead turtles during respirometric measurements.

**Fig. 4.**
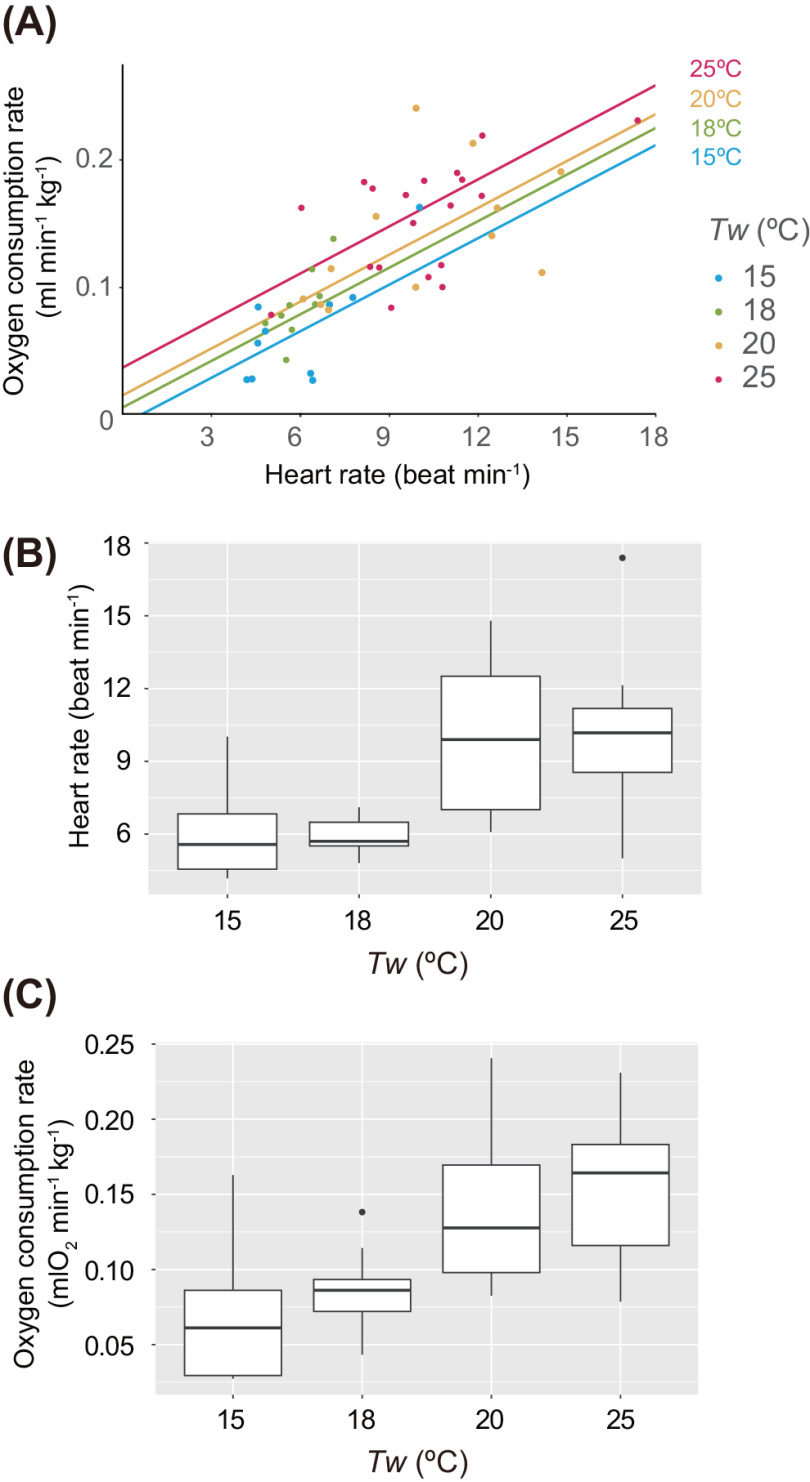
**(A) The relationship between average heart rate (*f*_H_: beats min^−1^) versus mass-specific oxygen consumption rate (**
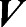
**_O_2__: mlO_2_** **min^−1^** **kg^−1^) for five loggerhead turtles.** Box plots showing *f*_H_ in B, 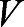
_O_2__ in C at each temperature during respirometric experiments. The model equation in A is 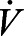
_O_2__=0.0124*f*_H_+0.0047*T*_w_ - 0.0791, and each line indicate blue in 15°, green in 18°C, orange in 20°C, and red in 25°C. Dots in A represent value during SDC (one dive+post surface interval) and routine dives.

Heart rates and oxygen consumption rates were measured in five loggerhead turtles using 15 indoor respirometric measurements (Table 1, Figs 1–3). Average dive durations were 27.1±20.1 min at 15°C, 27.7±19.2 min at 18°C, 28.2±9.6 min at 20°C, and 15.8±6.8 min at 25°C (Table 2). Activity ratios during measurement were 45.7±27.7% (range: 7.8–100.0%) (Fig. S2). The *f*_H_ during single dive cycles (SDCs) was 6.0±1.8 beats min^−1^ at 15°C (ten SDCs), 6.0±0.7 beats min^−1^ at 18°C (nine SDCs), 10.1±3.0 beats min^−1^ at 20°C (12 SDCs), and 10.0±2.5 beats min^−1^ at 25°C (19 SDCs) (Table 2, Fig. 4). The 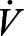
_O2_ during SDCs was 0.06±0.04 mlO_2_ min^−1^ kg^−1^ at 15°C (ten SDCs), 0.08±0.03 mlO_2_ min^−1^ kg^−1^ at 18°C (nine SDCs), 0.14±0.05 mlO_2_ min^−1^ kg^−1^ at 20°C (12 SDCs), and 0.15±0.04 mlO_2_ min^−1^ kg^−1^ at 25°C (19 SDCs) (Table 2, Fig. 4C). The linear mixed model (LMM) revealed that 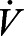
_O2_ was affected by *T*_w_ and *f*_H_ (Akaike information criterion; AIC=−185.51; Table 3). The model equation (Fig. 4A) of 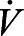
_O2_ (mlO_2_ min^−1^ kg^−1^) in loggerhead turtles, which included the effect of *T_w_* and *f*_H_ based on MLE, was calculated as:
(1)

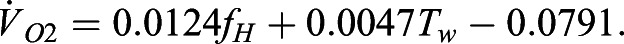
Mean surface and underwater heart rates are 10.5–19.0 beats min^−1^ and 4.6–6.5 beats min^−1^, respectively (Table 2).

**Table 1.**
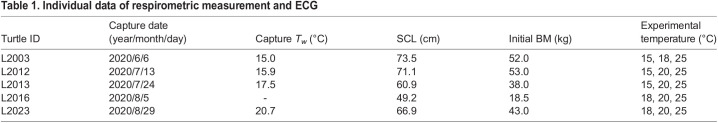
Individual data of respirometric measurement and ECG

**Table 2.**
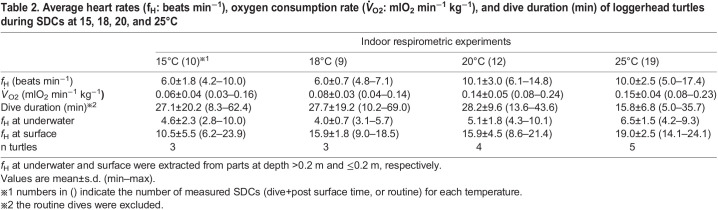
Average heart rates (f_H_: beats min^−1^), oxygen consumption rate (*V̇*_O2_: mlO_2_ min^−1^ kg^−1^), and dive duration (min) of loggerhead turtles during SDCs at 15, 18, 20, and 25°C

**Table 3.**
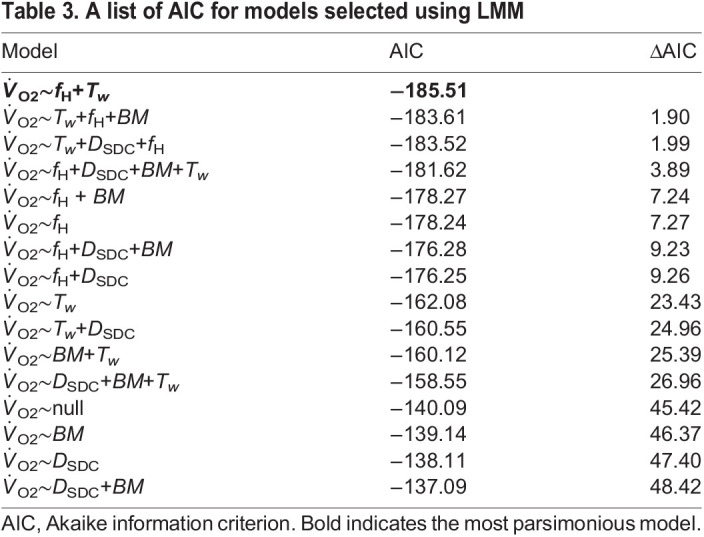
A list of AIC for models selected using LMM

## DISCUSSION

In the current study, a non-invasive method was used to simultaneously measure the heart rates of loggerhead turtles and oxygen consumption rates. We observed that 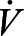
_O2_ was significantly affected by *T_w_* and *f*_H_. 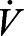
_O2_ estimated by Eqn 1 was close to values measured previously using respirometry (Kinoshita et al., 2018), thus Eqn 1 appears to be a reliable estimate of 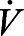
_O2_ provided that it does not deviate notably from the range of *f*_H_ measured in the current study (4.2–17.4 beats min^−1^). The oxygen pulse, i.e. the amount of oxygen consumed by the animal per heartbeat, should also be considered when investigating the relationship between oxygen consumption rates and heart rates. The heart rate method for estimating oxygen consumption rates is mainly based on Fick's convection equation, 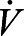
_O2_=*f*_H_ *V*_s_ (*C*_a_O_2_ – *C*
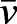
O_2_), for the cardiovascular system, where *V*_s_ is cardiac stroke volume, *C*_a_O_2_ is oxygen concentration of arterial blood, and *C*
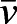
O_2_ is oxygen concentration of mixed venous blood. When the term *V*_s_ (*C*_a_O_2_ – *C*
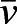
O_2_), which is known as the oxygen pulse, is constant or changes in a systematic manner, there will be a linear relationship between 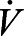
_O2_ and *f*_H_, and the regression passes through zero when the oxygen pulse is constant (Butler et al., 2004). Eqn 1 includes the effect of water temperature, which indicate oxygen pulse dependent on temperature.

Eqn 1 developed in the present study estimates fine-scale field oxygen consumption rates using *f*_H_ as a proxy; however, there are some limitations. First, as Eqn 1 was derived from juvenile loggerhead turtles in the summer, it does not account for factors such as seasonal conditions, which likely affect the relationship between 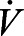
_O2_ and *f*_H_ in sea turtles (Southwood et al., 2003). Second, the experiments in this study were conducted in a filled water tank with 0.6 m depth. Loggerhead turtles have been reported to conduct deep (>200 m: Hatase et al., 2007; Narazaki et al., 2015; Polovina et al., 2003) and long dives (>300 min: Broderick et al., 2007; Hawkes et al., 2007; Hochscheid et al., 2007, Kinoshita et al., 2018). Thus, our experimental design did not consider physiological controls such as extreme diving bradycardia, which occurs only in the wild. For further detailed estimation of energy expenditure, additional experiments under field conditions are needed. Third, our experiments could not cover the upper limit in turtle heart rate during high activity. Leatherback turtles showed a 30% decrease in heart rate from the surface to underwater (Southwood et al., 1999). Our data also showed that the heart rate in loggerhead turtles decreased by 56–74% from surface to underwater (Table 3). Although the maximum value was not considered, most parts of the range in field heart rates will be covered. Lastly, *f*_H_ increased with increasing water temperature in the current study (Table 2) as it did in the previous study of loggerhead turtles (Hochscheid et al., 2002) and green turtles (*Chelonia mydas*) (Southwood et al., 2003). However, we did not consider differences in the relationship between heart rate and body temperature during acute heating and cooling, as reported for marine iguanas (Bartholomew and Lasiewski, 1965). In sea turtles, larger individuals have greater thermal inertia and slightly higher body temperatures than the ambient temperature (≤1.7°C) (Kinoshita et al., 2018; Sato, 2014), heart rates in response to acute changes in ambient temperature has not been clarified. Thus, the detailed correspondence between body temperature and heart rate should be investigated in the future.

There was a significant effect of water temperature on the relationship between heart rate and oxygen consumption rate in our study, as reported in marine iguanas (Butler et al., 2002). A further interesting question is whether there are population differences in these relationships. Loggerhead turtles used in our experiments originated from the North Pacific population and are known to exhibit lower thermal dependence (*Q*_10_=1.8) of metabolic rates than the Mediterranean population (*Q*_10_=5.4) (Hochscheid et al., 2004; Kinoshita et al., 2018). However, the range of heart rates of turtles in a tank observed previously (8.5–15.8 beats min^−1^; Hochscheid et al., 2002) was similar to that recorded in the current study (4.2–17.4 beats min^−1^). Thus, the thermal effect on the relationship between oxygen consumption rate and heart rate may differ among populations.

The range of FH measured in the current study (4.2–17.4 beats min^−1^) was similar to the heart rate observed in previous studies by implanting sensors into the body (6.4–10.9 beats min^−1^; Williams et al., 2019) and non-invasive techniques that ECG electrode patches were attached on the carapace (5.7–18.0 beats min^−1^; Sakamoto et al., 2021). Comparing the resting heart rates observed in the present study with those of other sea turtle species, the heart rates of green turtles (11.0–19.6 beats min^−1^; Okuyama et al., 2020; Southwood et al., 2003) were slightly higher than those of the loggerhead turtles. As the metabolic rates of green turtles and loggerhead turtles do not differ significantly in the North Pacific population (Kinoshita et al., 2021), the relationship between 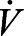
_O2_ and heart rate should differ between species. Thus, the equations should be evaluated on other species. Developing such equations explaining the relationship between oxygen consumption rate and heart rate for different sea turtle species, which exhibit different ecological traits, may produce valuable insights into the physiology of the species.

Using heart rate as an index, metabolic rates can be estimated on different time scales, from a few minutes to a day. Green et al. (2007) estimated the energetic costs of individual dives in macaroni penguins (*Eudyptes chrysolophus*), confirming that metabolic rate during diving is often substantially lower than assumed. Green et al. (2009) and Pelletier et al. (2008) estimated the average daily metabolic rates of macaroni penguins and common eiders (*Somateria mollissima*). In the present study, Eqn 1 was used to estimate the metabolic rate of loggerhead turtles calculated as the time spent on one dive and the post surface interval as one unit (SDCs; Fig. 2); thus, the estimation of oxygen consumption rate should be made on a time scale longer than SDC.

There are more than 60 studies on the relationship between heart rate and metabolic rate in mammals, birds, reptiles, and fishes (e.g. Armstrong, 1986; Butler et al., 2004; Green, 2011; Kooyman and Ponganis, 1994; Webb et al., 1998). In air-breathing diving animals, this relationship has been studied mainly in seabirds, pinnipeds, and dolphins (e.g. Butler et al., 1992; Green et al., 2001, 2005; Webb et al., 1998; Williams et al., 1991, 1993). To understand physiological adaptations of breath-holding diving animals, comprehensive studies across taxa are essential. Many studies have been biased toward endothermic animals, and there are few measurements while animals are diving. However, our study showed that heart rate correlates with oxygen consumption rate and water temperature in diving sea turtles. Eqn 1 allowed us to estimate the field energy expenditures of loggerhead turtles. It is now possible to acquire data on behaviour and heart rates of free-swimming sea turtles in the wild using automatic data logger release systems (Narazaki et al., 2009; Watanabe et al., 2004), which have been widely applied in behavioural tracking studies of migrating animals. Long-term field data will provide a more detailed information on the energy demand of each behaviour (e.g. swimming, resting, foraging), which will improve the understanding of physiological adaptations and decision-making behaviour in marine reptiles.

## MATERIALS AND METHODS

### Animals and study site

Juvenile loggerhead turtles were captured from June to August 2020 at a summer-restricted foraging ground (Narazaki et al., 2015). All experiments were conducted using five wild juvenile loggerhead turtles incidentally captured in set nets in the Sanriku coastal area (38°17′ to 39°28′N, 141°24′ to 142°00′E). Captured turtles were promptly transferred to tanks at the International Coastal Research Centre (ICRC), Atmosphere and Ocean Research Institute, The University of Tokyo (39°21′05 N, 141°56′04 E). Following the definition of Bolten, (1999), we measured straight carapace length (SCL in cm: from the notch to the tip of the carapace) and body mass (BM in kg). The turtles were kept in tanks for 32–74 days from the day of capture until the end of the experiments. After the experiments, all turtles were released promptly into the sea near the ICRC.

### ECG and behavioural data logger attachment

The ECG was measured using two types of ECG loggers (W400-ECG; Little Leonardo, Tokyo, Japan; cylindrical shape; 21 mm in diameter, 109 mm in length, 60 g in air, and ECG400-DT; Little Leonardo; cubical shape; 21 mm in width, 64 mm in length, 23 mm in height, 60 g in air). The ECG was recorded at 250 Hz. We also recorded acceleration (on the dorsoventral axis) and depth using a behavioural data logger (M190L-D2GT; Little Leonardo; 15 mm in diameter, 53 mm in length long, 18 g in air). Acceleration was recorded at 16 Hz, and depth at 1 Hz. The frontal areas of total package are 5.2 to 6.6 cm^2^.

Following the methods of Sakamoto et al. (2021) and Kinoshita et al. (2022), we non-invasively recorded ECG signals by attaching two electrode patches made of electro-conductive fabric (KNZ-ST50 shield cloth tape, Kyowa Harmonet Ltd., Kyoto, Japan), which was cut into squares (7×5 cm) and placed onto the plastron of each turtle (Fig. 1A). The electrodes were attached on the anterior and posterior parts of the plastron to measure ECG signals stably during respirometric measurements. To attach the electrodes to the plastron, we placed the turtles on a tire to immobilise them. The anterior electrode was used as the negative electrode and was located closed to the heart. After attaching the electrodes on the plastron, the electrodes were moistened with seawater to facilitate detection of the electrical signal on the surface of the plastron. The edges of the electrodes were glued onto the plastron using instant adhesive (Aron Alpha Jelly Extra, Konishi, Osaka, Japan). We insulated the outside of the electrode using waterproof adhesive plaster (Hydro Seal Extra Large, Johnson & Johnson, New Jersey, USA) (7×5 cm) and waterproof film (FC bosui film, Hakujuji Co., Ltd, Tokyo, Japan) (10×10 cm). To completely insulate the area between the plastron and the waterproof film, the edge of the waterproof film was sealed with epoxy (Bond quick 5; Konishi Co., Ltd, Osaka, Japan), which was allowed to dry for 5 min. The two lead wires extending from the ECG recorder were connected to the lead wires of the electrodes. On the edge of the carapace, we attached ECG and behavioural data loggers to measure ECGs and behavioural data (depth and accelerations) simultaneously (Fig. 1A). The recorders were fixed onto waterproof adhesive plaster using an instant adhesive and were then attached to the carapace. After attaching the recorders, the turtles were placed in the experimental water tank. The total time to attach the ECG recorder and data logger was approximately 40 min. After the respirometric experiments, all equipment was removed from the turtles, and data were downloaded.

### Respirometric measurements

The oxygen consumption rates of the turtles were measured in an open-flow respirometric experiment using an air chamber (see Kinoshita et al., 2018, Fig. 2A). We measured oxygen consumption rates at different water temperatures (15, 18, 20, and 25°C). This temperature range is similar to that experienced by loggerhead turtles in the North Pacific population throughout the year (Narazaki et al., 2015). The BM and SCL of the turtles ranged from 18.5 to 53.0 kg (40.9±12.5 kg) and from 49.2 to 73.5 cm (64.3±8.7 cm), respectively (Table 1). The turtles were kept in an experimental water tank (155×115×60 cm deep) filled with water at the respective experimental temperature for at least 5 days before the measurements were taken. These tanks were large enough for the turtles to move and make shallow dives (Fig. 2A). After attaching the ECG and behavioural loggers, the turtles were not disturbed for at least 12 h before the respirometric measurements to allow them to recover from handling effects. The water surface in the experimental tank was covered with a wooden board with an air hole. A 60.8 l air chamber (45×45×30 cm deep) was placed over this hole so that the turtle could only breathe within the chamber. A fan was mounted inside the air chamber to circulate the air. The flow rate of the respirometer was fixed at 3.0 l min^−1^ using a mass flow controller (Type HM1171A; Tokyo Keiso, Minato City, Tokyo, Japan), and the air in chamber was replaced approximately every 20.2 min. The air samples were passed through a dehumidifier to remove water vapor and were then pumped into an oxygen analyser (Xentra4100, Servomex Ltd, East Sussex, UK). Oxygen concentrations in the chamber were measured each second. The volume of oxygen consumed (mlO_2_) was calculated according to Kinoshita et al. (2018), and these values were averaged over 1 min and converted into a mass-specific rate representing the oxygen consumption rate (mlO_2_ min^−1^ kg^−1^). All results of oxygen consumption are presented at standard temperature (0°C) and pressure (1 atm) under dry conditions. All turtles were fed a diet of Japanese common squid (*Todarodes pacificus*) once every 3 days, and they were not fed on the respective day of the experiment. All respirometric measurements were conducted under indoor conditions.

### Calculating heart rates from ECG signals

The ECG recorder detected characteristic signals (QRS waves) associated with the turtle heartbeats. Following the methods of Sakamoto et al. (2021), the instantaneous heart rate was calculated as the reciprocal of the RR interval (Fig. 1B). The heart rate per minute was calculated as the median value of instantaneous heart rate per minute. For the signal processing part of the algorithm, we used the ECGtoHR program (Sakamoto et al., 2021), executed in IGOR Pro software version 8.04 (Wavemetrics, Portland, OR, USA). ECGtoHR removes noise from the ECG through a band-pass filter, detects R waves, and estimates the heart rate through the two parameters QRS wave frequency and maximum heart rate. The ECG, depth, and acceleration records were analysed using the Ethographer program package (Sakamoto et al., 2009) implemented in IGOR Pro.

### Relationships between oxygen consumption rate and heart rate

Loggerhead turtles hold their breath while diving and breathe at the surface after the dive, thus the time-series results of oxygen consumption rate lagged behind in the time-series results of heart rate during dives, which must be accounted for. The dive duration and the post-surface interval was defined as one SDC, and the oxygen consumptions during each SDC was reflected in the oxygen analyser after the dive (Fig. 2). As the average dive duration of turtles under these experimental conditions was 27.1–15.8 min at 15–25°C (Table 2), most of the air in the chamber could be replaced during the subsequent dive. Calculations of oxygen consumption rates were made assuming that turtles recover the oxygen consumed during the dive after surfacing. When the time between dives was very short and the SDCs could not be assigned unambiguously, the oxygen consumption rate and heart rate were calculated as a routine value indicating the average value over the measurement period (Fig. S1). The oxygen consumption rates (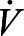
_O2_: mlO_2_ min^−1^ kg^−1^) and average heart rate (*f*_H_: beats min^−1^) corresponding to each SDC were calculated.

### Data analyses

To quantify activity ratio (%) during experiments, we analysed a longitudinal acceleration of turtles. If the standard deviation of longitudinal acceleration was less than 0.2 m s^−2^, the turtles were considered to be resting because this value was small and indicated no movement. To calculate surface and underwater heart rate, the turtle was defined as staying at the surface when the turtle reached a depth shallower than 0.2 m.

An LMM with a Gaussian distribution and identity link function was used to evaluate 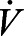
_O2_. In ectothermic animals, body temperature depends on ambient temperature, thus the effect of ambient temperature on the relationship between 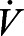
_O2_ and *f*_H_ must be considered (Butler et al., 2002). BM and duration of SDC (*D*_SDC_) were also taken into account as parameters that may affect 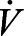
_O2_. The response variable was 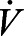
_O2_ and the explanatory variables were *T_w_*, BM, *f*_H_ and *D*_SDC_ during respirometric measurements, and the random effect was turtle ID in this study. The most parsimonious model was selected using the AIC. The coefficients for each term were estimated by the maximum likelihood estimation (MLE).

Statistical analyses were performed using R software version 3.3.2 (R Development Core Team, http://www.R-project.org). Shown are the means±standard deviation, unless otherwise indicated.

### Ethics statement

All experimental procedures were covered by the guideline of the Animal Ethic Committee of Atmosphere and Ocean Research Institute, the University of Tokyo, and the protocol of the study was approved by this committee (P20-11). This study was conducted as a part of a records and release program, in which loggerhead and green turtles caught by set nets through by-catch in the Sanriku Coast, were handed over to researchers by fishermen.

## Supplementary Material

Supplementary information
