# Supplementary material for: Heart rate as a proxy for estimating oxygen consumption rates in loggerhead turtles (Caretta caretta)
Source: Biol Open. 2022 Mar 31;11(3):bio058952. doi: 10.1242/bio.058952 (PMC8988048; doi:10.1242/bio.058952)
Supplement: Supplementary information [file biolopen-11-058952-s1.pdf]

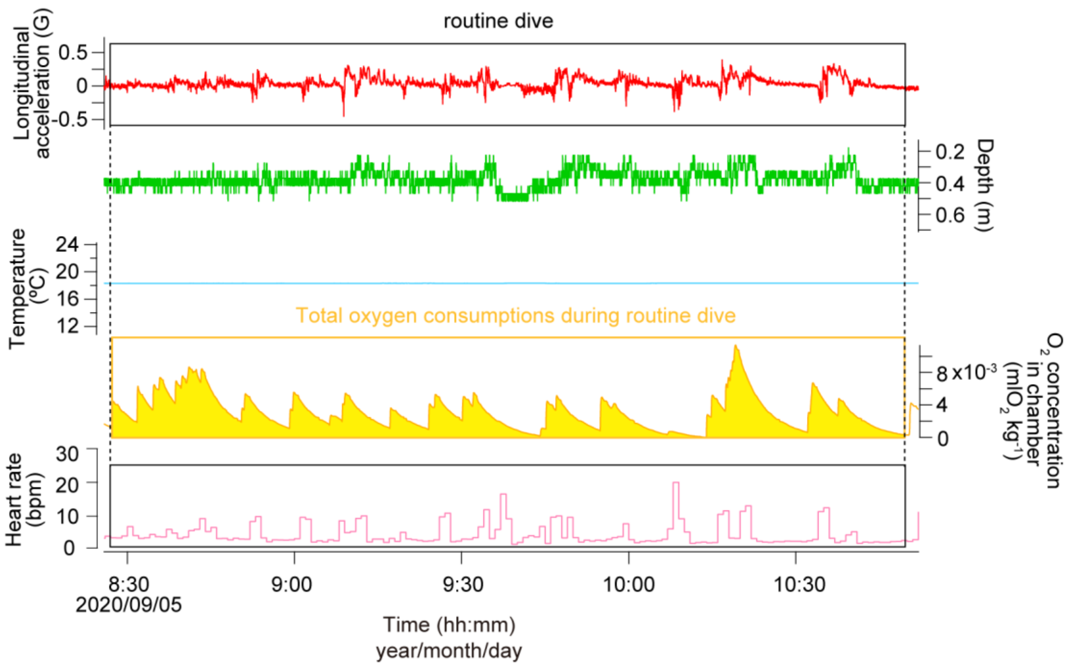

Fig. S1. Example of time-series data during routine dive by a loggerhead turtle (L2003).

Biology Open • Supplementary information

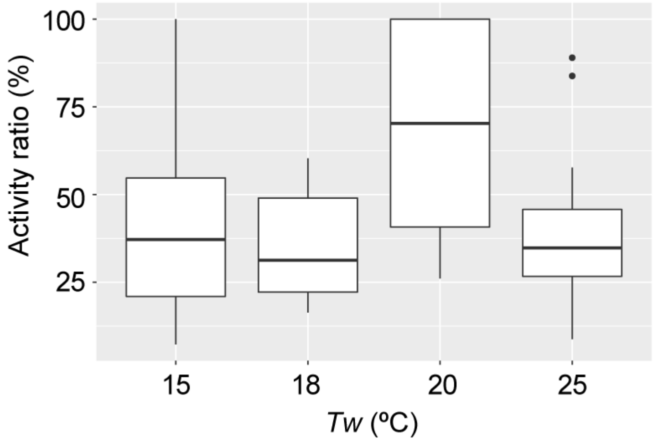

Fig. S2. Box plot showing activity ratio (%) at each temperature during respirometric measurements.

Biology Open • Supplementary information
